# Supplementary material for: PF4 Autoantibody Complexes Cause Activation of Integrins αIIbβ3 and αvβ3 and Possible Subsequent Thrombosis and Autoimmune Diseases
Source: Int J Mol Sci. 2025 Oct 22;26(21):10260. doi: 10.3390/ijms262110260 (PMC12610465; doi:10.3390/ijms262110260)
Supplement: Supplementary file 1 [file ijms-26-10260-s001.zip › ijms-3866123-supplementary.pdf]

Article

PF4 Autoantibody Complexes Cause Activation of Integrins αIIbβ3 and αvβ3 and Possible Subsequent Thrombosis and Autoimmune Diseases

Yoko K. Takada [1], Chun-Yi Wu [2] and Yoshikazu Takada [1,3,*]

[1] Department of Dermatology, University of California Davis School of Medicine, Sacramento, CA 95817, USA
[2] Department of Neurology, University of California Davis School of Medicine, Sacramento, CA 95817, USA; chywu@health.ucdavis.edu
[3] Department of Biochemistry and Molecular Medicine, University of California Davis School of Medicine, Sacramento, CA 95817, USA
* Correspondence: ytakada@health.ucdavis.edu

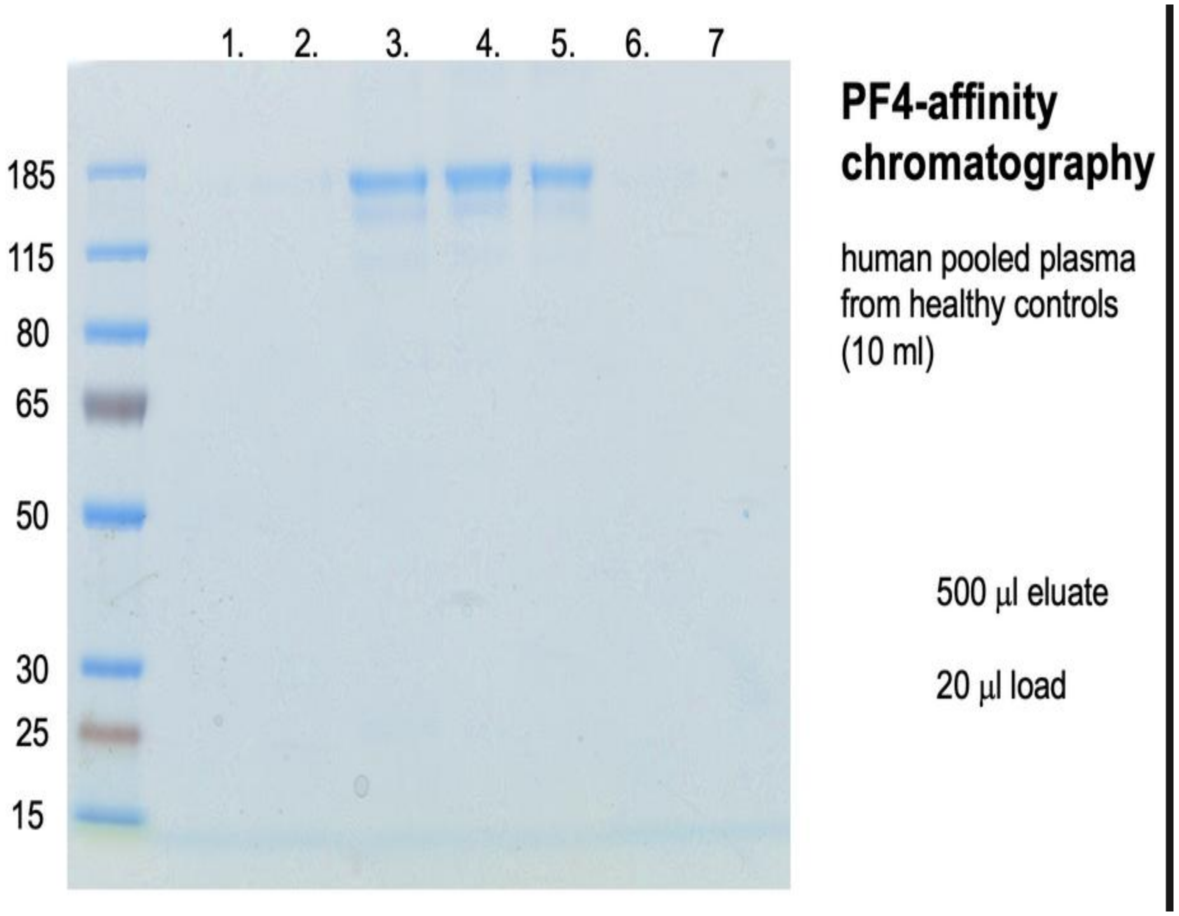

Accession	Descriptio	Coverage	Exp. q-vali	# Peptides	# Unique F	MW [kDa]	Biological	Cellular Cc	Molecular	Pfam IDs	Entrez Ger	Ensembl G	Gene Sym	Gene ID	Reactome
P0DOY3	Immunogl	96	0	11	5	11.3	signal tran	non-struct	other mol	Pf07654		ENSG0000	IGLC3	A0A075B6	Initial trigg
P01834	Immunogl	93	0	13	5	11.8	signal tran	non-struct	other mol	Pf07654			IGKC	A0A075B6	Initial trigg
P35527	Keratin, ty	80	0	31	30	62	cell organi	cytosol;cy	cytoskelet	Pf00038	3857	ENSG0000	KRT9	hsa:3857;	Formation
P01876	Immunogl	80	0	14	11	37.6									
P35908	Keratin, ty	77	0	39	31	65.4	cell cycle C	plasma m	bone, toot	Pf00038, P	3849	ENSG0000	KRT2	hsa:3849;	Formation
P01859	Immunogl	65	0	17	8	35.9									
P01871	Immunogl	61	0	25	5	49.4									
P04264	Keratin, ty	60	0	37	32	66	cell organi	non-struct	bone, toot	Pf00038, P	3848	ENSG0000	KRT1	B2RA01; h	Formation
P0DOX7	Immunogl	59	0	11	2	23.4	other biol	non-struct		Pf07654, P					
P01877	Immunogl	57	0	10	1	36.6									
P0DOX8	Immunogl	57	0	10	4	22.8	other biol	non-struct		Pf07654, P					
P01767	Immunogl	57	0	5	2	12.8	other biol	non-struct	other mol	Pf07686		ENSG0000	IGHV3-53	A0A087W	Initial trigg
A0A075B6	Immunogl	56	0	4	3	12.7	other biol	non-struct		Pf07686		ENSG0000	IGKV1-27	A0A075B6	
P0DOX5	Immunogl	53	0	19	11	49.3	other biol	non-struct		Pf07654, P					
P02768	Serum alb	53	0	27	25	69.3	stress resp	non-struct	nucleic aci	Pf00273	213	ENSG0000	ALB	E7ESS9; hs	Scavenging
O43866	CD5 antige	52	0	13	13	38.1	protein m	non-struct	other mol	Pf00530	922	ENSG0000	CD5L	A8K7M5; I	
P04433	Immunogl	52	0	3	3	12.6	other biol	non-struct	other mol	Pf07686		ENSG0000	IGKV3-11	P04433	Initial trigg
P0DOX2	Immunogl	51	0	13	4	48.9	other biol	non-struct		Pf00047, P					
P80748	Immunogl	49	0	3	3	12.4	other biol	non-struct	other mol	Pf07686		ENSG0000	IGLV3-21	A0A075B6	Initial trigg
P13645	Keratin, ty	48	0	27	25	58.8	cell organi	plasma m	bone, toot	Pf00038	3858	ENSG0000	KRT10	hsa:3858;	Formation
P01706	Immunogl	48	0	3	2	12.6	other biol	non-struct	other mol	Pf07686		ENSG0000	IGLV2-11	A0A075B6	Initial trigg
A0A0B4J1	Immunogl	47	0	5	5	13.2	other biol	non-struct	other mol	Pf07686		ENSG0000	IGHV3-72	A0A087W	
A0A0A0M	Immunogl	47	0	5	4	13	other biol	non-struct	other mol	Pf07686		ENSG0000	IGHV3-49	A0A0A0M	
P01619	Immunogl	47	0	4	2	12.5	stress resp	non-struct	other mol	Pf07686		ENSG0000	IGKV3-20	A0A0B4J1	Initial trigg
P08779	Keratin, ty	46	0	20	12	51.2	cell organi	plasma m	cytoskelet	Pf00038	3868	ENSG0000	KRT16	A8K488; h	Formation
A0M8Q6	Immunogl	46	0	4	1	11.2	signal tran	non-struct	other mol	Pf07654			IGLC7	A0A075B6	Initial trigg
P0DP08	Immunogl	44	0	4	1	13	other biol	non-struct	other mol	Pf07686			IGHV4-38-	P0DP08	
P01860	Immunogl	44	0	14	5	41.3									
P01714	Immunogl	44	0	3	3	12	other biol	non-struct	other mol	Pf07686		ENSG0000	IGLV3-19	A0A075B6	Initial trigg
P0DOX6	Immunogl	44	0	22	2	63.4	other biol	non-struct		Pf07654, P					
A0A0C4DH	Immunogl	43	0	3	2	12.5	other biol	non-struct		Pf07686		ENSG0000	IGKV1-8	A0A0C4DH	
P01824	Immunogl	42	0	4	1	13.9	other biol	non-struct	other mol	Pf07686		ENSG0000	IGHV4-39	A0A0A0M	Initial trigg
A0A0B4J1	Immunogl	41	0	5	5	12.9	other biol	non-struct	other mol	Pf07686		ENSG0000	IGHV3-15	A0A0B4J1	
P01768	Immunogl	41	0	5	2	12.9	other biol	non-struct	other mol	Pf07686		ENSG0000	IGHV3-30	A0A0B4J2	Initial trigg
P01699	Immunogl	38	0	2	1	12.2	other biol	non-struct	other mol	Pf07686		ENSG0000	IGLV1-44	A0A0B4J1	Initial trigg
P01764	Immunogl	38	0	4	1	12.6	other biol	non-struct	other mol	Pf07686		ENSG0000	IGHV3-23	A0A087W	Initial trigg
A0A075B6	Probable r	38	0	3	3	13.1	other biol	non-struct		Pf07686		ENSG0000	IGKV2D-24	A0A075B6	
P00739	Haptoglob	38	0	7	5	39		non-struct	other mol	Pf00089	3250	ENSG0000	HPR	hsa:3250;	Scavenging
P01780	Immunogl	38	0	5	3	12.9	other biol	non-struct	other mol	Pf07686		ENSG0000	IGHV3-7	A0A0B4J1	Initial trigg
P02538	Keratin, ty	37	0	23	3	60	cell organi	cytosol;cy	bone, toot	Pf00038, P	3853	ENSG0000	KRT6A	A4QPC1; I	Formation
A0A075B6	Immunogl	36	0	2	2	12.4	other biol	non-struct		Pf07686		ENSG0000	IGLV10-54	A0A075B6	
P01861	Immunogl	36	0	9	3	35.9									

Figure S1. Purification of anti-PF4 antibodies from human plasma and mass spectroscopic analysis. WT PF4 (1 mg) was immobilized to CH-Sepharose and remaining binding sites were blocked with Tris buffer. Pooled human plasma from healthy controls (10 ml) was incubated with PF4-Sepharose overnight at 4°C and extensively washed. Proteins were eluted with 0.1 M Tris-Glycine buffer, pH2.5, and neutralized with 1 M tris buffer. Twenty ul of each fraction (0.5 ml) was loaded to SDS-gel and stained. Fractions 3-5 were collected and desalted into 0.1% formic acid for mass spectroscopic analysis.
